# Supplementary material for: Etiology of acute febrile illness in the peruvian amazon as determined by modular formatted quantitative PCR: a protocol for RIVERA, a health facility-based case-control study
Source: BMC Public Health. 2023 Apr 11;23:674. doi: 10.1186/s12889-023-15619-6 (PMC10088183; doi:10.1186/s12889-023-15619-6)
Supplement: Supplementary file 1 — Supplementary Material 1 [file 12889_2023_15619_MOESM1_ESM.docx]

**Supplementary Table 1**. Real-time PCR assays on TaqMan Array Card

| **Disease** | **Agent** | **Primer** | **Source** |
| --- | --- | --- | --- |
| **Anaplasmosis** | **Anaplasma** | TTTTCTACAATACTTGTCATGAAAT | (1) modified |
|  |  | TTTTCTGCAATACTTGTCGTGAAAT |  |
|  |  | TCGGAATGTTACCGGGTGTT |  |
|  |  | FAM-CACTCCYGCCATTACCACCAGCCAAA-MGB |  |
| **Bartonellosis** | **Bartonella** | GGCTAAATIAGTAGTTGCAAAYGACA | (2) |
| **(Carrion's disease, cat scratch fever and trench fever)** | **(Bartonella bacilliformis, B. hensalae, B, quintana)** | GCTTCTGTTGCCAGGTG |  |
|  |  | FAM-ACCCCGCTTAAACCTGCGACG-MGB |  |
| **Borreliosis** | **Borrelia** | AGCYTTTAAAGCTTCGCTTGTAG | (3) modified |
| **(Relapsing fever and Lyme's disease)** |  | GCCTCCCGTAGGAGTCTGG |  |
|  |  | FAM-CCGGCCTGAGAGGGTGAWCGG-MGB |  |
| **Brucellosis** | **Brucella** | GCTTGAAGCTTGCGGACAGT | (2) |
|  |  | GGCCTACCGCTGCGAAT |  |
|  |  | FAM-AAGCCAACACCCGGCCATTATGGT-MGB |  |
| **Chikungunya** |  | TCACTCCCTGYTGGACTTGATAGA | (2) |
|  |  | TTGACGAACAGAGTTAGGAACATACC |  |
|  |  | FAM-AGGTACGCGCTTCAAGTTCGGCG-MGB |  |
| **Q fever** | **Coxiella burnetii** | CCGATCATTTGGGCGCT | (2) |
|  |  | CGGCGGTGTTTAGGC |  |
|  |  | FAM-TTAACACGCCAAGAAACGTATCGCTGTG-MGB |  |
| **Cytomegalovirus** | **Cytomegalovirus virus** | AGG TCTTCA AGG AAC TCA GCA AGA | (4) |
|  |  | CGG CAA TCG GTT TGT TGT AAA |  |
|  |  | GAG CCC GACTTT ACC ATC CA |  |
|  |  | CAG CCG GCG GTA TCG A |  |
|  |  | FAM-AMC CCG TCA GCC ATT CTCTCG GC-MGB |  |
|  |  | FAM-ACC GCA ACA AGA TT-MGB |  |
| **Epstein-Barr virus related disease: acute mononucleosis, B cell myeloproliferatve dysplasias** | **Epstein-Barr virus** | CGGAAGCCCTCTRGACTTC  CCCTGTTTATCCGATGGAATG  FAM-TGTACACGCACGAGAAATGCG-MGB | (5) |
| **Dengue Fever** | **Dengue** | GGATAGACCAGAGATCCTGCTGT | (2) |
|  |  | CAT TCC ATT TTC TGG CGT TC |  |
|  |  | CAA TCC ATC TTG CGG CGC TC |  |
|  |  | FAM-CAGCATCATTCCAGGCACAG-MGB |  |
|  | **Dengue serotype 1** | CAAAAGGAAGTCGYGCAATA | (6) |
|  |  | CTGAGTGAATTCTCTCTGCTRAAC |  |
|  |  | FAM-CATGTGGYTGGGAGCRCGC-MGB |  |
|  | **Dengue serotype 2** | CAGGCTATGGCACYGTCACGAT | (6) |
|  |  | CCATYTGCAGCARCACCATCTC |  |
|  |  | VIC-CTCYCCRAGAACGGGCCTCGACTTCAA-MGB |  |
|  | **Dengue serotype 3** | GGACTRGACACACGCACCCA | (6) |
|  |  | CATGTCTCTACCTTCTCGACTTGYCT |  |
|  |  | FAM-ACCTGGATGTCGGCTGAAGGAGCTTG-MGB |  |
|  | **Dengue serotype 4** | TTGTCCTAATGATGCTRGTCG | (6) |
|  |  | TCCACCYGAGACTCCTTCCA |  |
|  |  | VIC-TYCCTACYCCTACGCATCGCATTCCG-MGB |  |
| **Eastern Equine Encephailitis** | **Eastern Equine Encephalitis Virus -North American** | CGGGACCAAACWGTGGAG | (7) modified |
| **(EEV, North American variant and the Madariaga or South American variant)** | **Madriaga Virus (Eastern Equine Encephalitis-South America)** | CGGGATCAAACGGTCGAA |  |
|  |  | GGAACTTCCTTTCCTCCTTCA |  |
|  |  | FAM-CTTCTCTTCACCCCTTGGCCCTCC-MGB |  |
| **Ehrlichia** | **Ehrlichiosis** | TAC AAA GAT AAA ATC CTC ACA TTT | (8) |
|  |  | ATT CGT AGA TAT TAG GAG GAA CAC |  |
|  |  | FAM-CCT GTT TGC TCC CCA CGC TT-MGB |  |
| **Venezuelan hemorraghic fever** | **Guaranito** | GCT GCC GGA GCT GTC TGA | (9) |
|  |  | ATG GTG CGA GTT TGT GGA CTT |  |
|  |  | FAM-CAC CAA GTC CCT TAA AG-MGB |  |
| **Hepatitis B** | **Hepatitis B virus** | TGTCCTGGYTATCGCTGGAT | (10) modified |
|  |  | AAG AACCAAYAAGAAGATGAG |  |
|  |  | FAM-TGC GGCGTTTTATCAT-MGB |  |
| **Hepatitis delta** | **Heapatitis delta virus** | TGGCTCTCCCTTAGCCATCCGA | (11) |
|  |  | GGGTTTCCACTCACAGGTTTGC |  |
|  |  | VIC-CCGCGAGGAGGTGGAGATGCCAT-MGB |  |
| **Histoplasmosis** | **Histoplasma** | ACCCTTGTCTACCGGACCTGTT | (4) |
|  |  | TTTTGACTGGATTATTATCGCTCTYA |  |
|  |  | FAM-CGGTGAACGATTGGCGTCTGAGC-MGB |  |
| **Human Immunodeficinecy virus (Type 1)** | **HIV-1** | GCCTCAATAAAGCTTGCCTTGA | (12) |
|  |  | GGGCGCCACTGCTAGAGA |  |
|  |  | FAM-CCAGAGTCACACAACAGACGGGCACA-MGB |  |
| **Argentinian Hemmorhagic Fever** | **Junin** | CAT GGA GGT CAA ACA GCT TCC T | (9) |
|  |  | GCC TCC AGA CAT GGT TGT GA |  |
|  |  | VIC-ATG TCA TCG GAT CCT T-MGB |  |
| **Leishmaniasis** | **Leishmania** | AAGTGCTTTCCCATCGCAACT | (2) |
|  |  | GACGCACTAAACCCCTCAA |  |
|  |  | FAM-CGGTTCGGTGTGTGGCGCC-MGB |  |
| **Leptospirosis** | **Leptospira** | CCCTAIGGATCTGTRATCAACTA | (2) |
|  |  | GAA CTC CCA TTT CAG CGA TT |  |
|  |  | FAM-AA AGC CAG GAC AAG CGC CG-MGB |  |
| **Mayaro** | **Mayaro** | CCTTCACACAGATCAGAC | (13) |
|  |  | GCCTGGAAGTACAAAGAA |  |
|  |  | FAM-CATAGACRTCYTGATAGACTGCCACC-MGB |  |
| **Tuberculosis** | **Mycobacteria tuberculosis** | CCT ACT ACG ACC ACA TCA | (4) |
|  |  | CCG TAA ACA CCG TAG TTG |  |
|  |  | FAM-ATG TGC TCC TTG AGT TCG CCA T-MGB |  |
| **scrub typhus** | **Orientia tsutsugamushi** | AAC TGA TTTTAT TCA AAC TAA TGC TGC T | (14) |
|  |  | TAT GCC TGA GTA AGATAC RTG AAT RGA ATT |  |
|  |  | FAM-TGG GTA GCT TTG GTG GAC CGA TGT-MGB |  |
| **Oropouche** | **Oropouche virus** | TGATCCGGAGGCAGCATA | (14) |
|  |  | ACACCAGCATTGAGCACTTG |  |
|  |  | FAM-CCGTATCTAGCTTCAAATGCC-MGB |  |
| **Malaria** | **Plasmodium- genus specific** | GCTCTTTCTTGATTTCTTGGATG | (2) |
|  |  | AGCAGGTTAAGATCTCGTTCG |  |
|  |  | FAM-CACGAACTAAAAACGGCCAT-MGB |  |
| **Falciparum malaria** | **Plasmodium falciparum** | CGACTAGGTGTTGGATGAAAGTGTTAA | (15) modified |
|  |  | AACCCAAAGACTTTGATTTCTCATAA |  |
|  |  | FAM-CTAAAAGTCACCTCGAAAGATGA-MGB |  |
| **Plasmodium vivax** | **Plasmodium vivax** | GGATGGGAACTGCGAAGAAA | (16) |
|  |  | TCCGTCATTTCTTCTTCATACTGAG |  |
|  |  | FAM-ATCTGAGGCACTCGCT-MGB |  |
| **Rickettsiosis** | **Rickettsia** | AGCTTGCTTTTGGATCATTTGG | (2) |
|  |  | TTCCTTGCCTTTTCATACATCTAGT |  |
|  |  | FAM-CCTGCTTCTATTTGTCTTGC-MGB |  |
| **Salmonellosis** | **Salmonella-genus specific** | CTCACCAGGAGATTACAACATGG | (2) |
|  |  | AGCTCAGACCAAAAGTGACCATC |  |
|  |  | GGCAATTCGTTATTGGCGATA |  |
|  |  | CACGGTGACAATAGAGAAGACAACA |  |
|  |  | FAM-CACCGACGGCGAGACCGACTTT-MGB |  |
|  |  | VIC-CCTGGCGGTGGGTT-MGB |  |
| **Paratyphoid Fever** | **Salmonella paratyphoid A** | GCGGGGAACACGAATCATTC | (14) |
|  |  | GCATCATCGCGCATAGTGTC |  |
|  |  | FAM-CTCGGTTTATCCCCGCTGG-MGB |  |
| **Typhoid Fever** | **Salmonella enterica serovar Typhi** | CGCGAAGTCAGAGTCGACATAG | (2) |
|  |  | AAGACCTCAACGCCGATCAC |  |
|  |  | FAM-CAGCCTGCTCCAGAACA-MGB |  |
| **COVID-19** | **SARS-CoV-2** | GAC CCC AAA ATC AGC GAA AT | (17) |
|  |  | TCT GGT TAC TGC CAG TTG AAT CTG |  |
|  |  | TTA CAA ACA TTG GCC GCA AA |  |
|  |  | GCG CGA CAT TCC GAA GAA |  |
|  |  | FAM-ACC CCG CAT TAC GTT TGG TGG ACC-MGB |  |
|  |  | VIC-ACA ATT TGC CCC CAG CGC TTC AG-MGB |  |
| **Pneumocoocus** | **Streptococcus pneumoniae** | ACG CAA TCT AGC AGA TGA AGC A | (4) |
|  |  | TCG TGC GTT TTA ATT CCA GCT |  |
|  |  | FAM-CCGAAAACGCTTGATACAG-MGB |  |
| **Chagas disease** | **Trypanosoma cruzii** | GCA CTC GTC GCC TTT GTG | (18) |
|  |  | AGT TGA GGG AAG GCW TGA CA |  |
|  |  | ASTCGGCTGATCGTTTTCGA |  |
|  |  | AATTCCTCCAAGCAGCGGATA |  |
|  |  | FAM-CGAAGTCTGCCAACAACAC-MGB |  |
|  |  | VIC-CACACACTGGACACCAA-MGB |  |
| **West Nile Virus** | **West Nile Virus** | CAARGCCCARTGTCAGACCAC | (2) modified |
|  |  | TTTGTTMACCCAGTCCWCCTG |  |
|  |  | FAM-ACTCTGCGGAGAGTGCAGTC-MGB |  |
| **Yellow Fever** | **Yellow Fever Virus** | TGCTAATTGAGGTGCATTGG | (4) |
|  |  | CTGGTCAGTTCTCTGCTAATCG |  |
|  |  | FAM-TCTGCAAATCGAGTTGCTAGGCA-MGB |  |
| **Plague** | **Yersinia pestis** | CCACTGCAACGGCAACTCTT | (2) |
|  |  | TGTAATTGGAGCGCCTTCCT |  |
|  |  | FAM-TTGAACCAGCCCGCATCACTCTTACA-MGB |  |
| **Zika** | **Zika** | CCGCTGCCCAACACAAG | (14) |
|  |  | CCACTAACGTTCTTTTGCAGACAT |  |
|  |  | FAM-AGCCTACCTTGACAAGCAGTCAGACACTCAA-MGB |  |
| **Hepatitis E** | **Hepatitis E virus** | GGTGGTTTCTGGGGTGAC  AGGGGTTGGTTGGATGAA  FAM-TGATTCTCAGCCCTTCGC-MGB | (2) |
|  |  |  |  |

**Supplementary Table 2**. Real-time PCR Multiplex Assay for the Detection of SARS-CoV-2, Influenza A, and Influenza B.

| **Name** | **Description** | **Oligonucleotide Sequence (5' to 3')** |
| --- | --- | --- |
| Primers | | |
| **InfA-F1** | Influenza A Forward Primer 1 | CAA GAC CAA TCY TGT CAC CTC TGA C |
| **InfA-F2** | Influenza A Forward Primer 2 | CAA GAC CAA TYC TGT CAC CTY TGA C |
| **InfA-R1** | Influenza A Reverse Primer 1 | GCA TTY TGG ACA AAV CGT CTA CG |
| **InfA-R2** | Influenza A Reverse Primer 2 | GCA TTT TGG ATA AAG CGT CTA CG |
| **InfB-F** | Influenza B Forward Primer | TCC TCA AYT CAC TCT TCG AGC G |
| **InfB-R** | Influenza B Reverse Primer | CGG TGC TCT TGA CCA AAT TGG |
| **SC2-F** | SARS-CoV-2 Forward Primer | CTG CAG ATT TGG ATG ATT TCT CC |
| **SC2-R** | SARS-CoV-2 Reverse Primer | CCT TGT GTG GTC TGC ATG AGT TTA G |
| **RP-F** | RnaseP Forward | AGA TTT GGA CCT GCG AGC G |
| **RP-R** | RnaseP Revese | GAG CGG CTG TCT CCA CAA GT |
| Probes | | |
| **InfA-P** | Influenza A Probe | 5’-/5FAM/TGC AGT CCT **/**ZEN**/** CGC TCA CTG GGC ACG/3IABkFQ/-3’ |
| **InfB-P** | Influenza B Probe | 5’-/5YakYel/CCA ATT CGA/ZEN/ GCA GCT GAA ACT GCG GTG/3IABkFQ/-3’ |
| **SC2-P** | SARS-Cov-2 Probe | 5’-/5TexRd-XN/ATT GCA ACA/TAO/ ATC CAT GAG CAG TGC TGA CTC/3IAbRQSp/-3’ |
| **RP-P** | RnaseP Probe | 5’-/5CY5/TTC TGA CCT /TAO/ GAA GGC TCT GCG CG/3IAbRQSp/-3’ |

**Supplementary Table 3**. SARS-CoV-2, Influenza A, and Influenza B Positive Controls.

| **Influenza A** |
| --- |
| Quantitative Genomic RNA from Influenza A virus strain A/California/07/2009 pdm09 (H1N1) |
| ATCC Number: VR-1894DQ |
| **Influenza B** |
| Quantitative Genomic RNA from Influenza B virus strain B/Wisconsin/1/2010 BX-41A |
| ATCC Number: VR-1885DQ |
| **SARS-CoV-2** |
| Quantitative Synthetic Severe Acute Respiratory Syndrome-Related coronavirus (SARS-CoV-2) RNA: ORF, E, N. |
| ATCC Number: VR-3276SD |

1. Levin ML, Coble DJ, Ross DE. Reinfection with Anaplasma phagocytophilum in BALB/c mice and cross-protection between two sympatric isolates. Infect Immun. 2004;72(8):4723-30.

2. Liu J, Ochieng C, Wiersma S, Stroher U, Towner JS, Whitmer S, et al. Development of a TaqMan Array Card for Acute-Febrile-Illness Outbreak Investigation and Surveillance of Emerging Pathogens, Including Ebola Virus. J Clin Microbiol. 2016;54(1):49-58.

3. Parola P, Diatta G, Socolovschi C, Mediannikov O, Tall A, Bassene H, et al. Tick-borne relapsing fever borreliosis, rural senegal. Emerging infectious diseases. 2011;17(5):883-5.

4. Moore CC, Jacob ST, Banura P, Zhang J, Stroup S, Boulware DR, et al. Etiology of Sepsis in Uganda Using a Quantitative Polymerase Chain Reaction-based TaqMan Array Card. Clin Infect Dis. 2019;68(2):266-72.

5. Liu J, Gratz J, Amour C, Nshama R, Walongo T, Maro A, et al. Optimization of Quantitative PCR Methods for Enteropathogen Detection. PloS one. 2016;11(6):e0158199.

6. Waggoner JJ, Abeynayake J, Sahoo MK, Gresh L, Tellez Y, Gonzalez K, et al. Single-reaction, multiplex, real-time rt-PCR for the detection, quantitation, and serotyping of dengue viruses. PLoS neglected tropical diseases. 2013;7(4):e2116.

7. Maan S, Belaganahalli MN, Maan NS, Potgieter AC, Mertens PPC. Quantitative RT-PCR assays for identification and typing of the Equine encephalosis virus. Braz J Microbiol. 2019;50(1):287-96.

8. Chung IH, Austin AL, Kato CY. Development and validation of real-time PCR assays for the detection of Ehrlichia species and E. chaffeensis in clinical specimens. J Microbiol Methods. 2021;186:106225.

9. Trombley AR, Wachter L, Garrison J, Buckley-Beason VA, Jahrling J, Hensley LE, et al. Comprehensive panel of real-time TaqMan polymerase chain reaction assays for detection and absolute quantification of filoviruses, arenaviruses, and New World hantaviruses. Am J Trop Med Hyg. 2010;82(5):954-60.

10. Paraskevis D, Beloukas A, Haida C, Katsoulidou A, Moschidis Z, Hatzitheodorou H, et al. Development of a new ultra sensitive real-time PCR assay (ultra sensitive RTQ-PCR) for the quantification of HBV-DNA. Virol J. 2010;7(1):57.

11. Botelho-Souza LF, dos Santos Ade O, Borzacov LM, Honda ER, Villalobos-Salcedo JM, Vieira DS. Development of a reverse transcription quantitative real-time PCR-based system for rapid detection and quantitation of hepatitis delta virus in the western Amazon region of Brazil. J Virol Methods. 2014;197:19-24.

12. Joshi P, Maidji E, Stoddart CA. Inhibition of Heat Shock Protein 90 Prevents HIV Rebound*. Journal of Biological Chemistry. 2016;291(19):10332-46.

13. Friedrich-Janicke B, Emmerich P, Tappe D, Gunther S, Cadar D, Schmidt-Chanasit J. Genome analysis of Mayaro virus imported to Germany from French Guiana. Emerging infectious diseases. 2014;20(7):1255-7.

14. Rainey JJ, Siesel C, Guo X, Yi L, Zhang Y, Wu S, et al. Etiology of acute febrile illnesses in Southern China: Findings from a two-year sentinel surveillance project, 2017-2019. PloS one. 2022;17(6):e0270586.

15. Nijhuis RHT, van Lieshout L, Verweij JJ, Claas ECJ, Wessels E. Multiplex real-time PCR for diagnosing malaria in a non-endemic setting: a prospective comparison to conventional methods. European journal of clinical microbiology & infectious diseases : official publication of the European Society of Clinical Microbiology. 2018;37(12):2323-9.

16. Pholwat S, Liu J, Stroup S, Jacob ST, Banura P, Moore CC, et al. The Malaria TaqMan Array Card Includes 87 Assays for Plasmodium falciparum Drug Resistance, Identification of Species, and Genotyping in a Single Reaction. Antimicrob Agents Chemother. 2017;61(5).

17. CDC 2019-Novel Coronavirus (2019-nCoV)

Real-Time RT-PCR Diagnostic Panel

18. Piron M, Fisa R, Casamitjana N, Lopez-Chejade P, Puig L, Verges M, et al. Development of a real-time PCR assay for Trypanosoma cruzi detection in blood samples. Acta Trop. 2007;103(3):195-200.
